# Sentiment analysis of video danmakus based on MIBE-RoBERTa-FF-BiLSTM

**DOI:** 10.1038/s41598-024-56518-z

**Published:** 2024-03-09

**Authors:** Jianbo Zhao, Huailiang Liu, Yakai Wang, Weili Zhang, Xiaojin Zhang, Bowei Li, Tong Sun, Yanwei Qi, Shanzhuang Zhang

**Affiliations:** 1 School of Economics and Management, Xidian University, 266 Xifeng Road, Xi’an, 710071 China; 2 School of Telecommunications Engineering, Xidian University, 266 Xifeng Road, Xi’an, 710071 China

**Keywords:** Sentiment analysis, New word discovery, Sentiment annotation, Feature fusion, MIBE-RoBERTa-FF-BiLSTM, Computational neuroscience, Data acquisition, Data mining, Data processing, Machine learning, Computer science, Information technology

## Abstract

Danmakus are user-generated comments that overlay on videos, enabling real-time interactions between viewers and video content. The emotional orientation of danmakus can reflect the attitudes and opinions of viewers on video segments, which can help video platforms optimize video content recommendation and evaluate users’ abnormal emotion levels. Aiming at the problems of low transferability of traditional sentiment analysis methods in the danmaku domain, low accuracy of danmaku text segmentation, poor consistency of sentiment annotation, and insufficient semantic feature extraction, this paper proposes a video danmaku sentiment analysis method based on MIBE-RoBERTa-FF-BiLSTM. This paper constructs a “Bilibili Must-Watch List and Top Video Danmaku Sentiment Dataset” by ourselves, covering 10,000 positive and negative sentiment danmaku texts of 18 themes. A new word recognition algorithm based on mutual information (MI) and branch entropy (BE) is used to discover 2610 irregular network popular new words from trigrams to heptagrams in the dataset, forming a domain lexicon. The Maslow’s hierarchy of needs theory is applied to guide the consistent sentiment annotation. The domain lexicon is integrated into the feature fusion layer of the RoBERTa-FF-BiLSTM model to fully learn the semantic features of word information, character information, and context information of danmaku texts and perform sentiment classification. Comparative experiments on the dataset show that the model proposed in this paper has the best comprehensive performance among the mainstream models for video danmaku text sentiment classification, with an F1 value of 94.06%, and its accuracy and robustness are also better than other models. The limitations of this paper are that the construction of the domain lexicon still requires manual participation and review, the semantic information of danmaku video content and the positive case preference are ignored.

## Introduction

With the development of social media and video websites, user comments are rapidly increasing in quantity and diversity of forms. Danmaku is a new type of user-generated comment that scrolls in different positions on the video screen^1^, Users communicate with video producers and other users by posting danmakus containing emotions such as praise, sarcasm, ridicule, criticism, and compliments^2,3^. As an emerging information carrier, danmaku contains rich and real semantic information, which is an important corpus for sentiment analysis^4^, and the sentiment analysis of danmakus has important academic and commercial value. In the academic field, the sentiment analysis of danmakus helps to explore the emotional characteristics, expand the research field of sentiment analysis, and enrich the existing research theories and related technologies^5^; In the commercial field, danmakus sentiment analysis can effectively provide feedback of different users toward the video content, and help video platforms optimize the recommendation of video content and the management strategy of danmakus^6–9^; In the field of digital governance, danmakus sentiment analysis can be used to assess the abnormal emotion level of users, providing new methods for the detection of abnormal events on the Internet and the detection of users' mental health^10^.

Sentiment analysis is a specific application of natural language processing, machine learning and other technologies to extract feelings, emotions, opinions and attitudes in text data.Sentiment analysis based on user-generated texts can help governments, enterprises and individuals to understand the user's emotions and opinions, and provide support for decision-making in various fields. Existing sentiment analysis methods can be divided into lexicon-based methods, machine learning-based methods and deep learning-based methods, after a long period of development, these methods have been relatively mature and have achieved wide application in data fields such as movie reviews^11^, product reviews^12^, medical discussions^13^, and so on. As an emerging user-generated comment, the danmaku has its unique emotional and content characteristics compared to traditional comment data, and needs to be combined with the video content to analyze the potential meaning between the lines^7^. Danmakus are characterized by varying lengths and colloquial phrases, and there are a large number of self-composed words, abbreviated words, and popular new words in the content of danmkus, such as interactive phrases agreed with the video producers, which increase the difficulty of semantic understanding and emotional expression of danmaku texts, and bring a great challenge to the sentiment analysis^14^. Aiming at the new features of danmakus, scholars have carried out explorations and attempts of sentiment analysis. Traditional danmaku sentiment analysis methods mostly utilize sentiment lexicon and machine learning models to judge the sentiment tendency of danmakus, Cui et al.^15^ expanded the traditional sentiment lexicon, innovatively proposed a sentiment lexicon based on emoticons and tone words, and set a single sentiment threshold as a threshold interval, which extends the scope of neutral danmakus; Hong et al.^7^ extended the scope of neutral danmakus by improving the traditional k-means clustering algorithm and introducing Dynamic Time Warping (DTW) to calculate the distance between user emotion distributions and clustered the danmaku data. In recent years, with the development of neural networks, more scholars apply deep learning methods in the danmaku sentiment analysis tasks. Zhao et al.^16^ proposed a multi-head attention convolutional neural network (MH-ACNN) to analyze the sentiment of danmakus for their short content and incomplete contextual information; Hsieh and Zeng^17^ used ERNIE to characterize danmaku texts and then used BiLSTM to analyze danmakus.

Although existing researches have achieved certain results, they fail to completely solve the problems of low accuracy of danmaku text disambiguation, poor consistency of sentiment labeling, and insufficient semantic feature extraction^18^. Therefore, this paper proposes a danmaku sentiment analysis method based on MIBE-RoBERTa-FF-BiLSTM, which is based on the MIBE danmaku neologism recognition algorithm to discover non-regular popular words in danmaku texts, constructs a domain lexicon for Chinese lexicon, and at the same time, applies the Maslow's hierarchy of needs theory to guide sentiment annotation, and uses the RoBERTa pre-training model to adequately extract semantic and structural information from the danmaku semantic and structural information of danmaku texts, averaging the feature encoding of words after segmentation based on the feature fusion layer, and obtaining word encoding that retain the semantic information, using the bidirectional sequence modeling capability of BiLSTM model to effectively capture the contextual information of the danmaku text, better understand the semantic and dependency relationships in the danmaku text, and improve the model's emotional tendency classification ability and generalization ability. The purpose of this paper is to comprehensively improve the effectiveness of Chinese danmaku sentiment analysis model from 3 aspects of new words discovery, sentiment annotation, and conflicts between Chinese word segmentation methods and pre-training model tokenizer methods, assistant theoretical breakthroughs, with a view to help video platforms optimize video content recommendations and assess users’ abnormal sentiment levels, et al.

This paper is structured as follows: "Related work" section, introduces the work related to video danmaku sentiment analysis; "Model design" section, danmaku sentiment analysis model design; "Experiments and results" section, experiments and results analysis; "Discussions" secttion, discusses textual research innovations, limitations, and future perspectives; "Conclusion" section, summarizes the research in this paper.

## Related work

Currently, the main video danmaku sentiment analysis methods include sentiment lexicon-based methods, machine learning-based methods, and deep learning-based methods, and the specific research progress is as follows:

### Lexicon-based danmaku sentiment analysis

Sentiment analysis method for danmaku based on sentiment lexicon, by constructing a sentiment lexicon containing positive and negative sentiment words, segmenting danmakus and matching them with the sentiment lexicon, using an algorithm to classify danmakus and calculating the sentiment values^19^. Zheng et al.^20^ used a method based on the semantic weighting of sentiment words to calculate danmakus’ sentiment values and categorize danmakus; Wang and Xu^21^ combined the universal sentiment lexicons and danmaku multidimensional sentiment lexicons to construct an exclusive sentiment lexicon for danmakus, and then combined the time series to study the change trend of danmakus after calculating the sentiment values; Li et al.^22^ combined the lexicon-based danmaku sentiment categorization method with the plain Bayesian method; Liu et al.^23^ and Zeng et al.^24^ analyzed the influence of danmaku emotions on consumers' purchase intention by classifying danmaku emotions based on an emotion lexicon; Jin et al.^25^ fused the improved word forest with the HowNet similarity computation algorithm, and categorized the multidimensional lexicon according to the seven human emotion dimensions, and measured the danmaku emotion values by the improved emotion value computation method.

Sentiment lexicon-based approaches rely too much on the quality and coverage of the sentiment lexicon, with limited scalability and objectivity. The meanings of sentiment words may vary with context and time, increasing the limitations of the lexicon^26^; In addition, the development of sentiment lexicons and judgment rules requires a great deal of manual design and priori knowledge. The difficulties of sentiment annotation make the quality of the lexicons uneven. The development of social media has led to the continuous emergence of new online terms in danmakus, and the sentiment lexicon is difficult to adapt to the diversity and variability of danmakus timely. Therefore, the effect of danmaku sentiment analysis methods based on sentiment lexicon isn’t satisfactory.

*RQ1*: How to enable annotators to accurately annotate danmaku sentiment tendencies quickly, simply, reasonably and consistently understood in conjunction with video content?

*RQ2*: How to efficiently discover non-regular popular words in danmaku texts, cut danmaku texts into more reasonable words, and improve the quality of word embeddings in danmaku sentiment classification models?

### Machine learning-based danmaku sentiment analysis

A machine learning based approach for danmaku sentiment analysis, preprocessing danmaku data, constructing datasets, selecting and vectorizing text features, and training machine learning models for danmaku sentiment classification. Yang Deng et al.^6^ proposed a Multi-Topic Emotion Recognition (MTER) algorithm based on the Hidden Dirichlet Distribution (LDA) model for video clips, which utilizes the implicit emotional dependencies of the words in each danmaku to compute the emotion values and compute the emotion vectors; Jun Xu et al.^27^ utilized the Simple Bayes and Maximum Entropy methods to improve the accuracy of comment sentiment classification by selecting semantically inclined words as feature terms, correctly handling negations, and using binary values as feature term weights; Shang et al.^28^ used SnowNLP in conjunction with LDA topic modeling for sentiment analysis of multi-class review data; Hu et al.^29^ combined stuttering disambiguation and polynomial plain Bayes to construct a classifier for comment sentiment classification.

Machine learning-based methods require a large amount of labeled data and appropriate feature extraction methods, and have higher requirements for classification models; at the same time, this type of methods cannot fully utilize contextual information of the context, which affects the accuracy of classification to a certain extent.

*RQ3*: How to extract semantic and structural information of danmaku text words in different contexts to effectively capture contextual information?

### Deep learning-based danmaku sentiment analysis

Deep learning-based approach for danmaku sentiment analysis by multilayer neural networks. Ye et al.^30^ proposed a data collection algorithm based on hotspot detection and a model to analyze danmaku sentiment based on danmaku sentiment lexicon and convolutional neural network; Wang et al.^31^ categorized the four emotions of happiness, anger, sadness, and joy through the danmaku sentiment data analysis model based on the BiLSTM model; Bai et al.^32^ compared how models such as logistic regression, support vector machines, and recurrent neural networks predict the positive or negative sentiment of danmaku's comments and reflect it in video sentiment curves; Li and Mou^33^ used ERNIE and TextCNN to fuse danmaku's textual and temporal features, and then use BILSTM to perform sentiment analysis on the feature-fused vectors; Li et al.^34^ constructed a seed sentiment lexicon to compute danmaku text similarity for very short danmaku text sentiment recognition, and borrow BILSTM combined with BERT model for regular text danmaku sentiment recognition. Li et al.^35^ used the XLNet model to evaluate the overall sentiment of danmaku comments as pessimistic or optimistic.

Deep learning-based methods have stronger feature learning capabilities, reducing the cost of building and selecting features^36^, but the method needs to be based on a large amount of data, and is prone to data sparsity and overfitting problems in the case of small datasets^37^; Mainstream text pre-training models use different segmentation methods, BERT and XLNet use WordPiece, RoBERTa uses Byte-Pair Encoding, and the use of Chinese segmentation tools can easily lead to the fact that there is no difference between the Chinese segmented corpus and the pre-segmented one after tokenizer processing, which loses semantic information and results in model performance degradation.

*RQ4*: How to effectively extract the word information after Chinese word segmentation when the Chinese word segmentation method is inconsistent with the tokenizer method when using the text pre-training model?

Combining the above studies, this paper proposes a danmaku sentiment analysis model based on MIBE-RoBERTa-FF-BiLSTM, a neologism recognition algorithm based on mutual information (MI) and branch entropy (BE) to identify non-regular popular words in danmaku texts, so as to quickly construct a domain lexicon for accurate Chinese word segmentation. At the same time, Maslow's hierarchy of needs theory is applied to guide consistent sentiment annotation, and Roberta's pre-training model, feature fusion layer and Bilstm model are used in combination to adequately extract semantic features of danmaku texts, which effectively improves the ability to analyze the sentiment tendency of danmaku texts.

### Ethical approval

This article does not contain any studies with human participants performed by any of the authors.

## Model design

**Figure 1:**
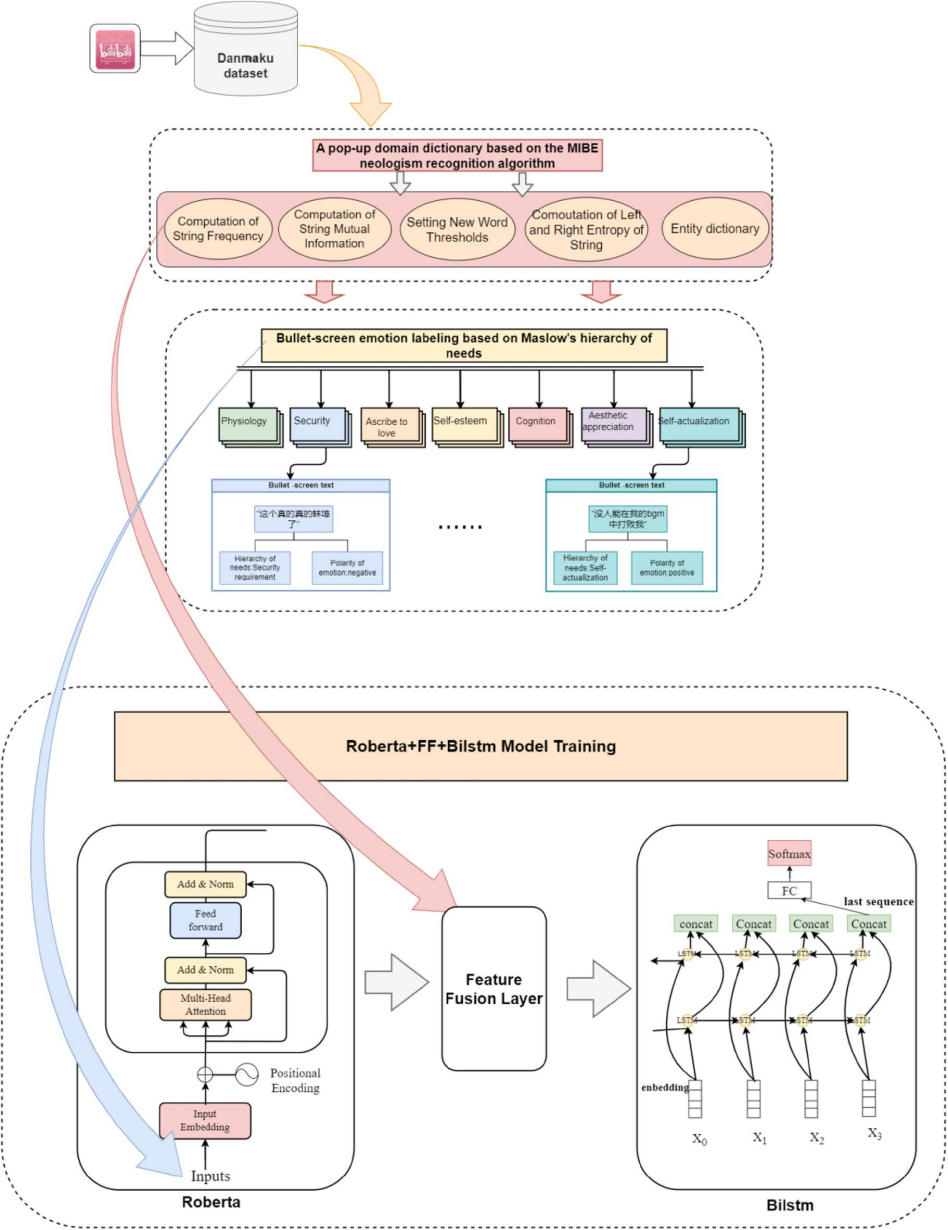
Framework diagram of the danmaku sentiment analysis method based on MIBE-Roberta-FF-Bilstm.

This paper proposed a danmaku sentiment analysis method based on MIBE-RoBERTa-FF-BiLSTM, which specifically includes the construction of danmaku domain lexicon based on MIBE neologism recognition algorithm, danmaku text sentiment annotation based on Maslow's hierarchy of needs theory, and RoBERTa-FF-BiLSTM sentiment analysis model. The overall framework of the research methodology is shown in Fig. 1.

### Danmaku domain lexicon construction based on MIBE neologism recognition algorithm

Danmaku texts are often highly colloquial, with a large number of non-standard popular words, such as "破防了", originally a game term referring to the use of skills to break through defenses, but in the context of the Internet, it expresses empathy for sensationalism or exciting videos; "蚌埠住了" is harmonized as "绷不住了", which describes the impact on the senses and can't help laughing or having an emotional meltdown. These Internet buzzwords contain rich semantic and emotional information, but are difficult to be recognized by general-purpose lexical tools. danmaku domain lexicon can effectively solve this problem by automatically recognizing and manually annotating these neologisms into the lexicon, which in turn improves the accuracy of downstream danmaku sentiment analysis task.

This paper proposes a danmaku neologism recognition algorithm based on mutual information and branch entropy. The algorithm incrementally expands candidate words by calculating mutual information with right neighbors, identifying potential neologisms. A screening process using branch entropy eliminates words with smaller left and right neighbor entropy, along with deactivated words at the beginning and end of candidate neologisms. The algorithm automatically creates a danmaku domain lexicon by comparing recognized neologisms with existing lexical phrases in the corpus. This approach enhances lexicon precision, capturing specific language nuances in danmaku interactions. After comparing the recognized new words with the existing phrases in the corpus, the meaningful new words are filtered out, and the danmaku domain lexicon is formed automatically, and at the same time, the new words are added to the participle lexicon to improve the quality of the participle lexicon. The specific steps are as follows:

Mutual Information (MI) is a common method used to measure the degree of co-occurrence of two variables in a corpus, and the larger the value indicates that the degree of dependence and the relationship between the two objects is also stronger. In the process of neologism discovery, it can be counted whether the probability of co-occurrence of two or more characters in the corpus reaches a certain threshold. The calculation method is shown in (1):

1 $$MI(A,B)={\text{log}}2\frac{p(A,B)}{p(A),p(B)}$$

where p(A) and p(B) denote the probability of word or phrase A and B appearing individually in the corpus set, respectively, p(A,B) denotes the joint probability of A and B co-occurring in the corpus set, and ${\text{MI}}\left({\text{A}},{\text{B}}\right)$ denotes the degree of dependency between A and B. If ${\text{MI}}\left({\text{A}},{\text{B}}\right)>0$, the probability of A and B co-occurring is greater than the product of the probability of each of them occurring individually, it means that the two may be related to each other, and the larger the value of MI, it means that the stronger the correlation between the two, and the more likely that they may form a new vocabulary; and if ${\text{MI}}\left({\text{A}},{\text{B}}\right)<0$,it means that A and B are independently distributed in the corpus set.

Branch Entropy (BE) is used to measure whether the neighboring characters of a candidate new word are stable enough, the larger the value indicates that the neighboring characters of the candidate new word contain more information, and the higher the probability of forming a word. The left neighbor entropy, right neighbor entropy are calculated as shown in (2) and (3).

2 $${H}_{L}(W)=-{\sum }_{{W}_{l}\in {S}_{l}}p\left({W}_{l}|W\right)\mathit{log}p\left({W}_{l}|W\right)$$

3 $${H}_{R}(W)=-{\sum }_{Wr\in Sr}p({W}_{r}|W)\mathit{log}p({W}_{r}|W)$$

${{\text{S}}}_{{\text{l}}}$ is the set of left neighbors of candidate word W, ${{\text{S}}}_{{\text{r}}}$ is the set of right neighbors of candidate word W, ${\text{P}}({{\text{W}}}_{{\text{L}}}|{\text{W}})$ denotes the conditional probability that ${{\text{W}}}_{{\text{L}}}$ is the left neighbor of candidate word W, ${\text{P}}({{\text{W}}}_{{\text{R}}}|{\text{W}})$ denotes the conditional probability that ${{\text{W}}}_{{\text{R}}}$ is the right neighbor of candidate word W, and the computational equations for ${\text{P}}({{\text{W}}}_{{\text{L}}}|{\text{W}})$ and ${\text{P}}({{\text{W}}}_{{\text{R}}}|{\text{W}})$ are shown in (4) and (5).

4 $$p\left({W}_{L}|W\right)=\frac{N\left({W}_{L},W\right)}{N\left(W\right)}$$

5 $$p({W}_{R}|W)=\frac{N(W,{W}_{R})}{N(W)}$$

where ${\text{N}}({{\text{W}}}_{{\text{L}}},{\text{W}})$ denotes the number of times ${{\text{W}}}_{{\text{L}}}$ and Wco-occur and N(W) denotes the number of times W occurs. Similarly, ${\text{N}}({{\text{W}}}_{{\text{R}}},{\text{W}})$ denotes the number of times ${{\text{W}}}_{{\text{R}}}$ and Wappear together. Taking the Internet buzzword "心态崩了" as an example, the prerequisite for it to become a separate word is that the words "心态" and "崩了" co-occur in the corpus at a high frequency, and the randomness of the words distributed around it should be strong enough.

The word-by-word expansion of the uncut danmaku corpus is mainly applied to the recognition of neologisms of three or more characters. Taking the neologism "蚌埠住了" as an example, after the binary neologism "蚌埠" is counted, the mutual information between "蚌埠" and "住" is calculated by shifting to the right and finally expanding to "蚌埠住了". By calculating the mutual information and eliminating the words with low branch entropy and removing the first and last deactivated words, the new word set is obtained after eliminating the existing old words. In addition, this method achieves dynamic evolution of the danmaku lexicon by excluding new words that may contain dummy words at the beginning and end, and adding new words to the lexicon without repetition after comparing them with those in the danmaku lexicon. This approach improves the quality of word splitting and solves the problems of unrecognized new words, repetitions, and garbage strings. In this paper, a total of 9851 neologisms from three to seven dollars were identified by the above method, and after manual checking and reviewing, 2610 neologisms with realistic significance were finally retained to constitute the danmaku neologism lexicon, and Table 1 shows the statistics of some of the neologisms and their manual annotations.

**Table 1:** Danmaku neologisms meaning.

No	Neologism	Meaning
1	trigger warning	Self-wording: used as a reminder to the rest of the audience that what's coming up next is very exciting, so be prepared
2	I can't laugh anymore	Self-wording: indicate that the content of the video is so hilarious that you can't help but let out a chuckle
3	I'll definitely do it next time	The interaction between the viewer and the producer of the video is similar to the interactive behavior of "liking", when the producer of the video asks the viewer to like the video, the viewer expresses approval of the author, or teases or mocks the poor quality of the video
4	My youth is making a comeback	Abbreviation: youth is back, used to express surprise and admiration when a remembered thing or person returns in a different state of appearance
5	have no martial ethics	Internet buzzwords: indicate that the behavior of the characters in the video or the content of the video caught off guard, completely unexpected, mostly used to tease the video producer or the commercials inserted in the video mockery, you need to combine with the content of the video to make judgments

### Danmaku emotion annotation based on Maslow's hierarchy of needs theory

The danmaku texts contain internet popular neologisms, which need to be combined with the video content to analyze the potential meanings between the lines, and the emotion annotation is difficult. Currently, it is widely recognized that individuals produce emotions influenced by internal needs and external stimuli, and that when an individual's needs are met, the individual produces positive emotions, otherwise negative emotions are generated^38^. Therefore, this paper decomposes and maps the hierarchy of needs contained in danmaku content, which can be combined with video content to make a more accurate judgment of danmaku emotions. This paper adopts Maslow's hierarchy of needs theory, which includes seven levels of physiological, safety, belonging and love, self-esteem, cognitive, aesthetic, and self-actualization needs, for guiding the labeling of danmaku emotions. This paper invited 10 senior Bilibili users to watch the video and then use the method to label the sentiment polarity of danmaku text. Compared with the labeling without using the method, the difficulty of the labeling is greatly reduced, and the speed and accuracy of the labeling are significantly improved. Examples of the labeling results are shown in Table 2.

**Table 2:** Examples of Danmaku emotion annotation based on Maslow's hierarchy of needs.

No	Danmaku texts	Hierarchy of needs	Sentimental polarity	Traditional annotation	Maslow annotation
1	Play your own script anyway	Self-actualization needs	Positive	Difficult	Easy
2	Magnificent, to the boss knelt down	Aesthetic needs	Positive	Medium	Easy
3	I really want a hug, even for strangers	Belonging and love needs	Negative	Difficult	Easy
4	This is really, really Bengbu	Security needs	Negative	Difficult	Easy
5	I'm sorry I was born a man	Self-esteem needs	Negative	Medium	Easy

**Figure 2:**
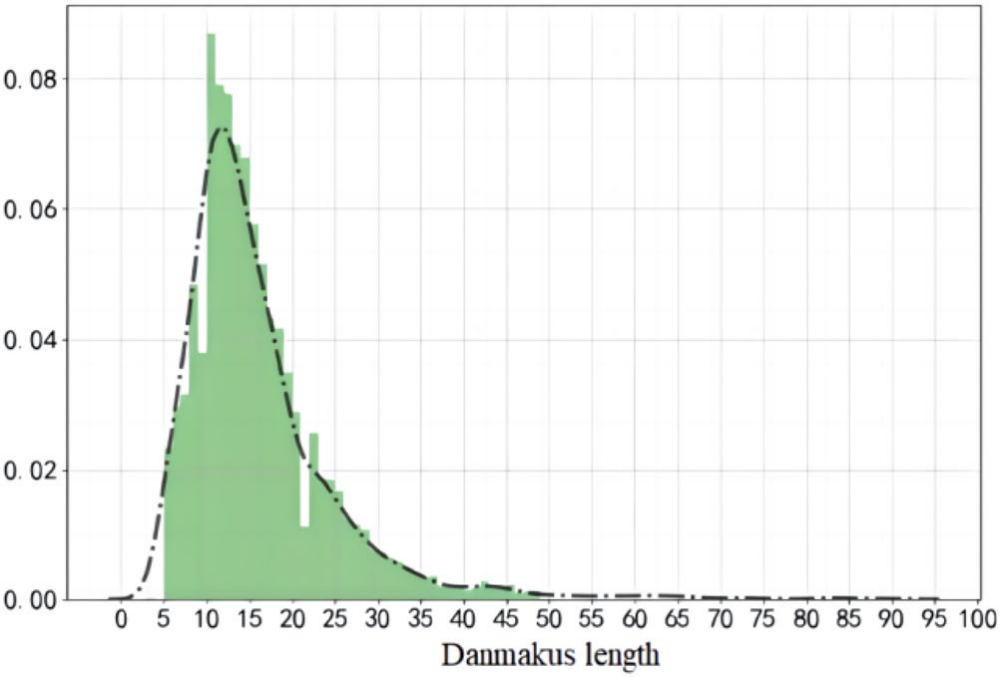
Danmakus length distribution.

The semantic structure of danmaku text is loosely structured and contains a large number of special characters, such as numbers, meaningless symbols, traditional Chinese characters, or Japanese, etc. These symbols, which contain only a small amount of emotional information, will bring noise to the neural network, so this paper eliminates these redundant information through regular expressions.Meanwhile, this paper visualizes and analyzes the danmaku length, as shown in Fig. 2, and finds that the danmaku length is mainly distributed between 5 and 45 characters, so this paper excludes the danmaku texts whose lengths are more than 100 or less than 5.

### RoBERTa-FF-BiLSTM sentiment analysis model

**Figure 3:**
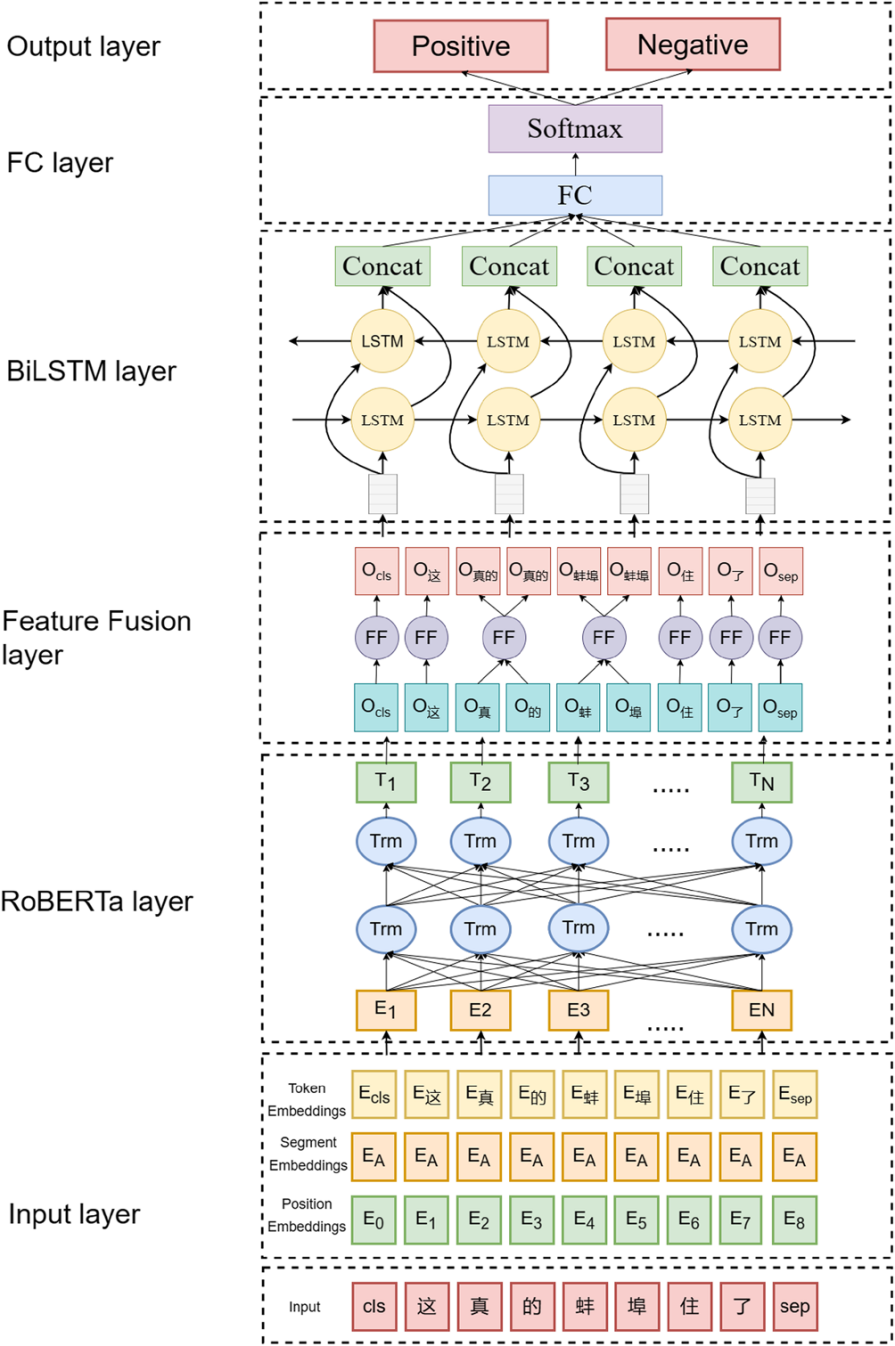
RoBERTa-FF-BiLSTM danmaku text sentiment recognition model.

This paper uses the RoBERTa model to pre-train and extract the deep semantic information in danmaku texts, and the corresponding word vectors of the words in the Chinese phrases after word splitting are fused with the features, so that the output word embedding vectors of the RoBERTa model can contain more fine-grained information of the Chinese corpus, and then the information is inputted into the BiLSTM model to deal with the danmaku text's contextual information for sentiment classification. The model structure is shown in Fig. 3.

**Figure 4:**
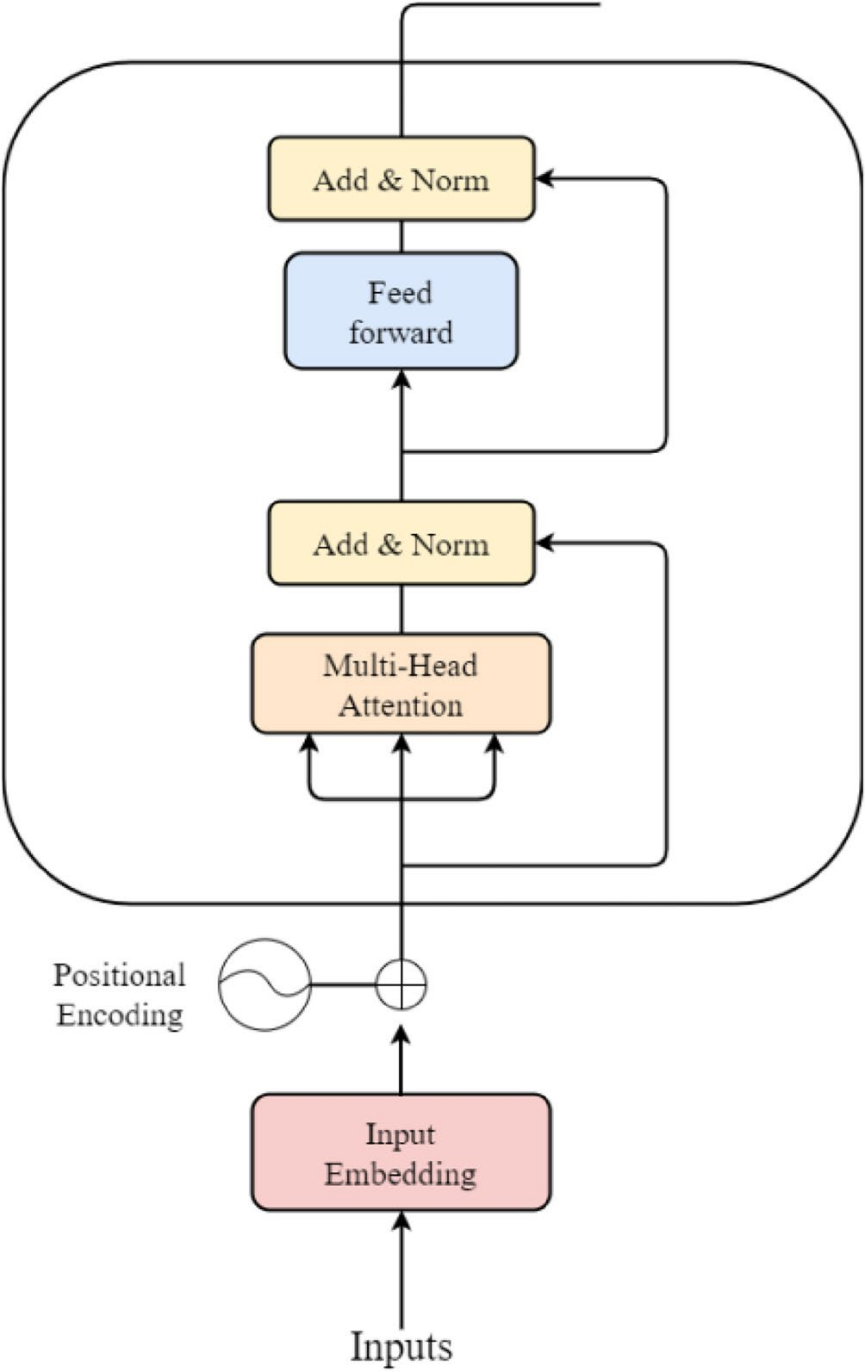
Structure of Transformer Encoder Decode.

By increasing the randomness and diversity of the pre-training data, RoBERTa can better learn the deep semantic information of the text and improve the accuracy of the downstream text categorization task. RoBERTa model is a bidirectional Transformer encoder based on the Bidirectional Encoder Representations from Transformers (BERT) model, which mainly utilizes Transformer-Encoder for computation. Each Encode module is composed of three parts: multi-head attention mechanism, residual connection and layer normalization, and feed-forward neural network, as shown in Fig. 4:

In Fig. 4, the word vectors are obtained by transforming the words in the input text through the one-hot encoding representation, and the positional encoding indicates the relative or absolute position of the word in the sequence, and the word embedding vectors generated by superposition of the two are used as the inputs X. The multi-head attentionmechanism, as a self-attention mechanism, is the core unit in the Transformer encoder, which uses multiple independent Attention modules to perform concurrent operations on the input information, and its operational formula is shown in (6):

6 $$Attention\left(Q,K,V\right)=Softmax\left(\frac{Q{K}^{T}}{\sqrt{{d}_{k}}}\right)V$$

where $\left\{Q,K,V\right\}$ is the input matrix and ${d}_{k}$ is used as the input matrix dimension. The multi-head attention mechanism delivers the resulting hidden vector twice to the next layerafter the multi-head self-attention computation: residual connectionand layer normalization. The layer normalization transforms the input into mean–variance and the residual connection adds the input X with the result obtained from the nonlinear transformation as the output term. The inputs are then operated on by the two fully connected layers of the feedforward neural network, applying the formula shown in (7):

7 $${W}_{e}=max(0,X{W}_{0}+{b}_{0}){W}_{0}^{\mathrm{^{\prime}}}+{b}^{\mathrm{^{\prime}}}$$

where $\left\{{W}_{e},{W}_{0}{\prime}\right\}$ is the weight matrix of the two connected layers and $\left\{{b}_{e},{b}_{0}{\prime}\right\}$ is the bias term of the two connected layers. After each word embedding vector in the input layer is encoded by the RoBERTa layer encoding operation, a bidirectional correlation between word embedding vectors can be established, which enables the model to learn the semantic features contained in each word embedding vector in different contexts. For example, "这真的蚌埠住了" or "太感人了蚌埠住了", in which the word "蚌埠" expresses very different semantics in different contexts, RoBERTa pre-training model can be based on large-scale text pre-training to derive a common linguistic expression, and then fine-tuned to transfer the learned knowledge to different downstream tasks. Based on this, when dealing with the problem of polysemous words in different contexts, the RoBERTa pre-training model obtains the semantic features in different contexts by pre-training the textual information, and then inputs them into the BiLSTM model for sentiment classification.

**Figure 5:**
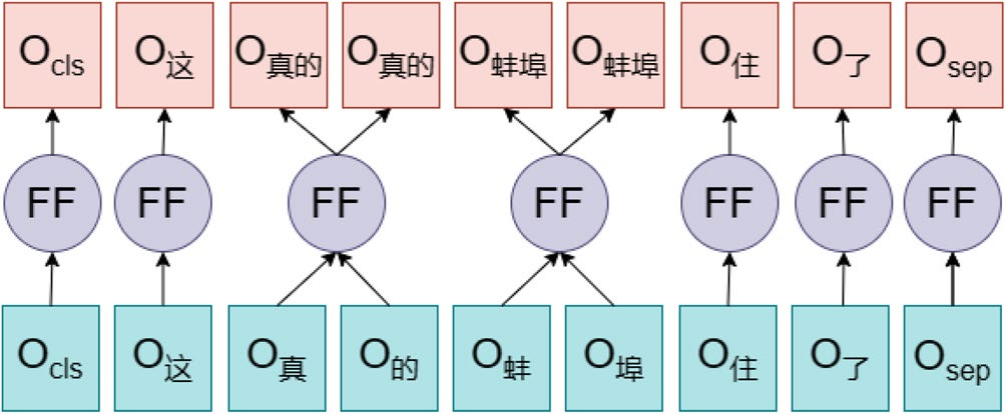
Logical structure diagram of Feature Fusion Layer.

Before inputting to the BiLSTM layer, the word embedding vectors output from RoBERTa need to be inputted to the Feature Fusion Layer for processing, and the corresponding word vectors of the words in the lexicon are fused with the features, so that the word embedding vectors output from the RoBERTa model can contain more fine-grained Chinese corpus information. In the feature fusion layer, the jieba thesaurus is first used to segment the text, for example, in the sentence "This is really Bengbu lived", the jieba segmentation tool divides this sentence into ['this', 'really', 'Bengbu', 'lived', 'had']. In this paper, the number of words contained in each word in this sentence is counted to get the vector of [1,1,1,2,2]. When the word embedding vector output by RoBERTa is obtained, this paper averages the words in the same word and fills them into the original position, thus realizing the purpose of feature fusion, the logical structure is shown in Fig. 5.

**Figure 6:**
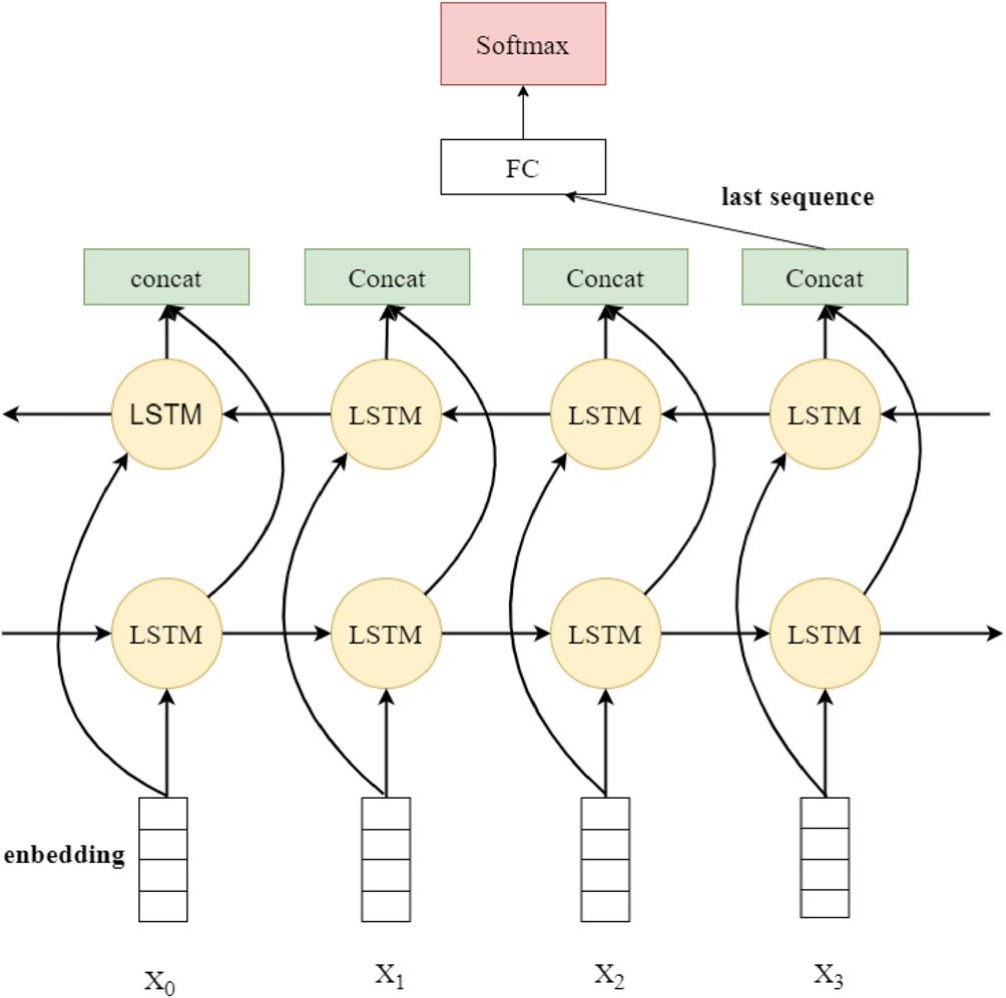
Logical structure of BiLSTM-bidirectional LSTM.

In a unidirectional LSTM, neuron states are propagated from the front to the back, so the model can only take into account past information, but not future information^39^, which results in LSTM not being able to perform complex sentiment analysis tasks well. For example, in the case of the danmaku "专家说的挺好的,下次别说了", the literal message above expresses positive appreciation, but it can only be judged in the context of the semantics of the following sentence that it is the danmaku sender's derision and flirtation, and that the danmaku does not express a positive emotion. To solve this situation it is necessary to introduce a bidirectional LSTM.The BiLSTM model of the Bi-Long Short-Term Memory Network BiLSTM is composed of a forward-processing sequence LSTM with a reverse-processing sequence LSTM as shown in Fig. 6. This paper establishes a BiLSTM layer after the RoBERTa layer, and utilizes BiLSTM to extract features from the contextual information of the input texts, which effectively makes up for the shortcomings of the RoBERTa layer that lacks the consideration of contextual information.

The BiLSTM model for extracting features for contextual information takes the final output matrix ${\text{C}}\in {{\text{R}}}^{{\text{n}}}$ 与 of the RoBERTa layer with weights ${{\text{W}}}_{{\text{a}}}\in {{\text{R}}}^{{{\text{d}}}_{{\text{a}}}\times {\text{n}}}$ and adds it as an input to the bidirectional LSTM. The computational public is shown in (8):

8 $${a}_{i}={g}_{1}\left({W}_{a}{C}_{i}+{b}_{a}\right)$$

where n is the dimension of the feature vector obtained after pre-training in the sentence; ${{\text{a}}}_{{\text{i}}}\in {{\text{R}}}^{{{\text{d}}}_{{\text{a}}}}$; ${{\text{b}}}_{{\text{a}}}$ is bias vector and the dimension is ${{\text{d}}}_{{\text{a}}}$; Bidirectional LSTM is computed on the hidden layers in two different directions, and the hidden vectors $\overrightarrow{{\text{h}}}$, $\overleftarrow{{\text{h}}}$ of the last layer of the forward and backward LSTMare merged and used as the output, the output vector ${{\text{V}}}_{{\text{i}}}$ at moment i. The computational formula is shown in (9):

9 $${V}_{i}=\overrightarrow{{h}_{i}}+\overleftarrow{{h}_{i}}$$

where $\overrightarrow{{{\text{h}}}_{{\text{i}}}}\in {{\text{R}}}^{{{\text{d}}}_{{\text{h}}}}$, $\overleftarrow{{{\text{h}}}_{{\text{i}}}}\in {{\text{R}}}^{{{\text{d}}}_{{\text{h}}}}$, The output is passed through a fully connected layer, and the Tanh function is used as the activation function ${{\text{g}}}_{2}$ to add nonlinear factors for hidden layer computation, where the computational metrics are shown in (10):

10 $${h}_{i}^{d}={g}_{2}\left({W}_{h}^{d}+U{h}_{i-1}^{d}+{b}_{h}^{d}\right)$$

where ${{\text{W}}}_{{\text{h}}}^{{\text{d}}}\in {{\text{R}}}^{{{\text{d}}}_{{\text{a}}}\times {{\text{d}}}_{{\text{h}}}}$ is the weight matrix of a corresponding to the index of the dth; U is the weight matrix of the output b of the corresponding i-1 moment of the hidden layer; ${\text{d}}\in \left\{\mathrm{0,1}\right\}$ denotes the different directions in the hidden layer; and ${{\text{b}}}_{{\text{b}}}^{{\text{d}}}\in {{\text{R}}}^{{{\text{d}}}_{{\text{h}}}}$ is the bias vector corresponding to the index of the *d*th. Afterwards, all ${{\text{h}}}^{{\text{d}}}$’s in the hidden layer are combined to form the final sentence-level feature vector ${\text{H}}$. The feature vector ${\text{H}}$ is fed into the fully connected layer and the ${\text{ReLU}}$ activation function is used. The output of the fully connected layer is used as the input of the output layer, and the classification is performed using the ${\text{Softmax}}$ function, whose probability formula is shown in (11):

11 $${\text{p}}\left({\text{y}}|\mathrm{ H},{{\text{W}}}_{{\text{S}}},{{\text{b}}}_{{\text{s}}}\right)={\text{Softmax}}\left({{\text{W}}}_{{\text{s}}}\cdot {\text{H}}+{{\text{b}}}_{{\text{s}}}\right)$$

where ${{\text{W}}}_{{\text{S}}}\in {{\text{R}}}^{|{\text{s}}|\times |{\text{l}}|}$ and ${{\text{b}}}_{{\text{s}}}\in {{\text{R}}}^{|{\text{l}}|}$ is the parameter of the output layer; $|{\text{l}}|$ is the number of propensity categories.

## Experiments and results

In order to verify the effectiveness and superiority of the danmaku sentiment analysis method based on MIBE-RoBerta-FF-BiLSTM proposed in this paper, this paper carries out comparative experiments on the self-constructed danmaku text dataset of "Bilibili Must-Watch List and Top Video Danmaku Sentiment Dataset", and the experimental results show that the performance of the method proposed in this paper is optimal, and the following describes the dataset used in this paper, the evaluation indexes, parameter settings, the comparative experimental method, as well as the results of the experiments and analysis, respectively.

### Dataset

This paper collect danmaku texts from Bilibili through web crawler, and construct a "Bilibili Must-Watch List and Top Video Danmaku Sentiment Dataset". Bilibili is one of the first video platforms in China to implement the danmaku mechanism, with rich video themes and a broad user base, and its user-generated danmaku is highly representative. This paper focuses on crawling the must-swipe list and the head video danmaku under each topic, covering 18 topics, such as dramas and documentaries. The danmakus of 50 negative sentiment videos were crawled specifically, totaling 4.1 million entries. After inviting 10 senior bilibili users to watch the video, Maslow's hierarchy of needs theory was used to guide the danmaku sentiment annotation, and 10,000 positive and negative sentiment danmaku each were obtained, with a total of 20,000 pieces of data, and the dataset was divided into a training set and a test set according to 8:2. The participle lexicon uses the jieba segmentation tool and the MIBE-based danmaku domain lexicon containing 2610 neologisms.

### Evaluation indicators

This paper uses confusion matrix to statistically analyze and evaluate the model's sentiment classification results, using TP to denote danmaku samples with positive actual sentiment and positive prediction, FP to denote danmaku samples with negative actual sentiment predicted as positive, TN to denote danmaku samples with negative actual sentiment and negative prediction, and FN to denote danmaku samples with positive actual sentiment but negative prediction. FN denotes danmaku samples whose actual emotion is positive but the prediction result is negative. Accuracy (ACC), precision (P), recall (R), and reconciled mean F1 are used to evaluate the model, and the formulas are shown in (12)–(15).

12 $$Acc=\frac{TP+TN}{TP+FP+TN+FN}$$

13 $$P=\frac{TP}{TP+FP}$$

14 $$R=\frac{TP}{TP+FN}$$

15 $$F1=\frac{2\times P\times R}{P+R}$$

### Parameter settings

To evaluate the performance of the method proposed in this paper on the danmaku sentiment analysis task, experiments were conducted on NVIDIA GeForce RTX3060 using Python 3.8 and PyTorch framework. Chinese-RoBerta-WWM-EXT, Chinese-BERT-WWM-EXT and XLNet are used as pre-trained models with dropout rate of 0.1, hidden size of 768, number of hidden layers of 12, max Length of 80. BiLSTM model is used for sentiment text classification with dropout rate of 0.5, hidden size of 64, batch size of 64, and epoch of 20. The model is trained using Adam optimizer with a learning rate of 1e−5 and weight decay of 0.01.

### Comparative experimental method

In order to validate the effectiveness of the danmaku sentiment analysis model (MIBE-RobERTa-FF-BiLSTM) proposed in this paper, we compare its performance with the mainstream baseline models for sentiment analysis in recent years on a self-constructed dataset under the same experimental environment: BiLSTM, SVM and BernoulliNB models with Word2Vec vectorization, and BERT-BiLSTM, XLNET-BiLSTM, RoBERTa-BiLSTM, RoBERTa-LSTM, RoBERTa-RNN, RoBERTa-TextCNN models with the training paradigm of “pre-trained model + neural network classifier”; Using jieba lexicon and embedding FF feature fusion layer, compare models’ performance; Jieba-BERT-FF-BiLSTM,Jieba-XLNET-FF-BiLSTM,Jieba-RoBERTa-FF-BiLSTM,Jieba-RoBERTa-FF-LSTM,Jieba-RoBERTa-FF-RNN,Jieba-RoBERTa-FF-TextCNN;Adding danmaku neologism lexicon based on MIBE neologism recognition algorithm to jieba lexicon and embedding FF feature fusion layer, compare models’ performance; MIBE-BERT-FF-BiLSTM, MIBE-XLNET-FF-BiLSTM, MIBE-RoBERTa-FF-BiLSTM, MIBE-RoBERTa-FF-LSTM, MIBE-RoBERTa-FF-RNN, MIBE-RoBERTa-FF-TextCNN.

### Experimental results and analysis

The results of the comparison experiment are shown in Table 3.

**Table 3:** Performance statistics of the sentiment analysis models.

Model	Accuracy (%)	Recall (%)	F1 (%)
SVM	88.50	88.46	88.68
BernoulliNB	74.55	61.90	71.24
BiLSTM	74.05	74.08	74.05
BERT-BiLSTM	93.78	95.94	93.81
XLNet-BiLSTM	93.15	94.79	93.08
RoBERTa-BiLSTM	93.85	96.19	93.87
RoBERTa-LSTM	93.73	95.79	93.67
RoBERTa-RNN	93.65	95.79	93.69
RoBERTa-TextCNN	93.73	95.99	93.70
Jieba-BERT-FF-BiLSTM	93.85	95.94	93.84
Jieba-XLNet-FF-BiLSTM	93.20	95.34	93.20
Jieba-RoBERTa-FF-BiLSTM	93.88	95.69	93.88
Jieba-RoBERTa-FF-LSTM	93.80	95.79	93.77
Jieba-RoBERTa-FF-RNN	93.75	95.74	93.81
Jieba-RoBERTa-FF-TextCNN	93.83	95.89	93.82
MIBE-BERT-FF-BiLSTM	93.93	96.54	93.89
MIBE-XLNet-FF-BiLSTM	93.33	94.64	93.25
MIBE-RoBERTa-FF-BiLSTM	94.10	95.84	94.06
MIBE-RoBERTa-FF-LSTM	93.98	95.14	93.95
MIBE-RoBERTa-FF-RNN	94.03	95.24	94.04
MIBE-RoBERTa-FF-TextCNN	93.85	97.34	93.84

**Figure 7:**
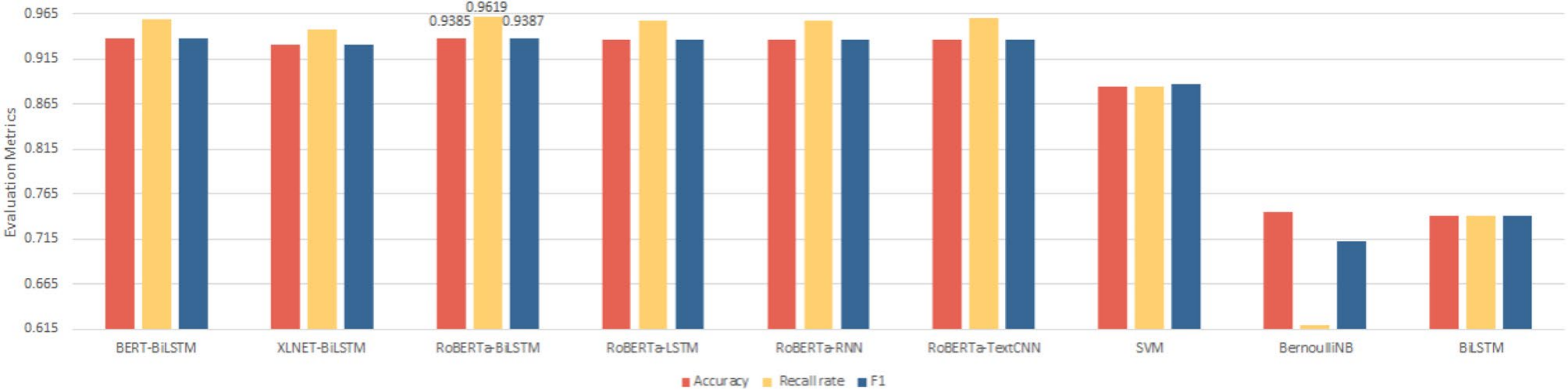
Performance statistics of mainstream baseline model for sentiment analysis.

**Figure 8:**
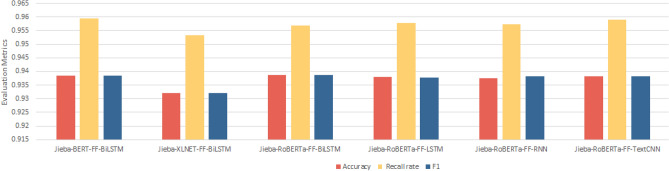
Performance statistics of mainstream baseline model with the introduction of the jieba lexicon and the FF layer.

**Figure 9:**
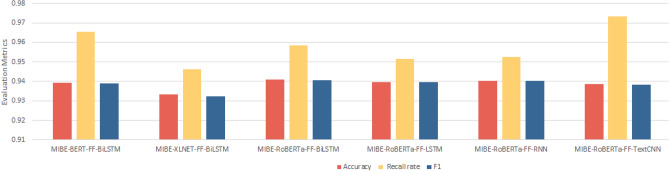
Performance statistics of mainstream baseline model with the introduction of the MIBE-based lexicon and the FF layer.

**Figure 10:**
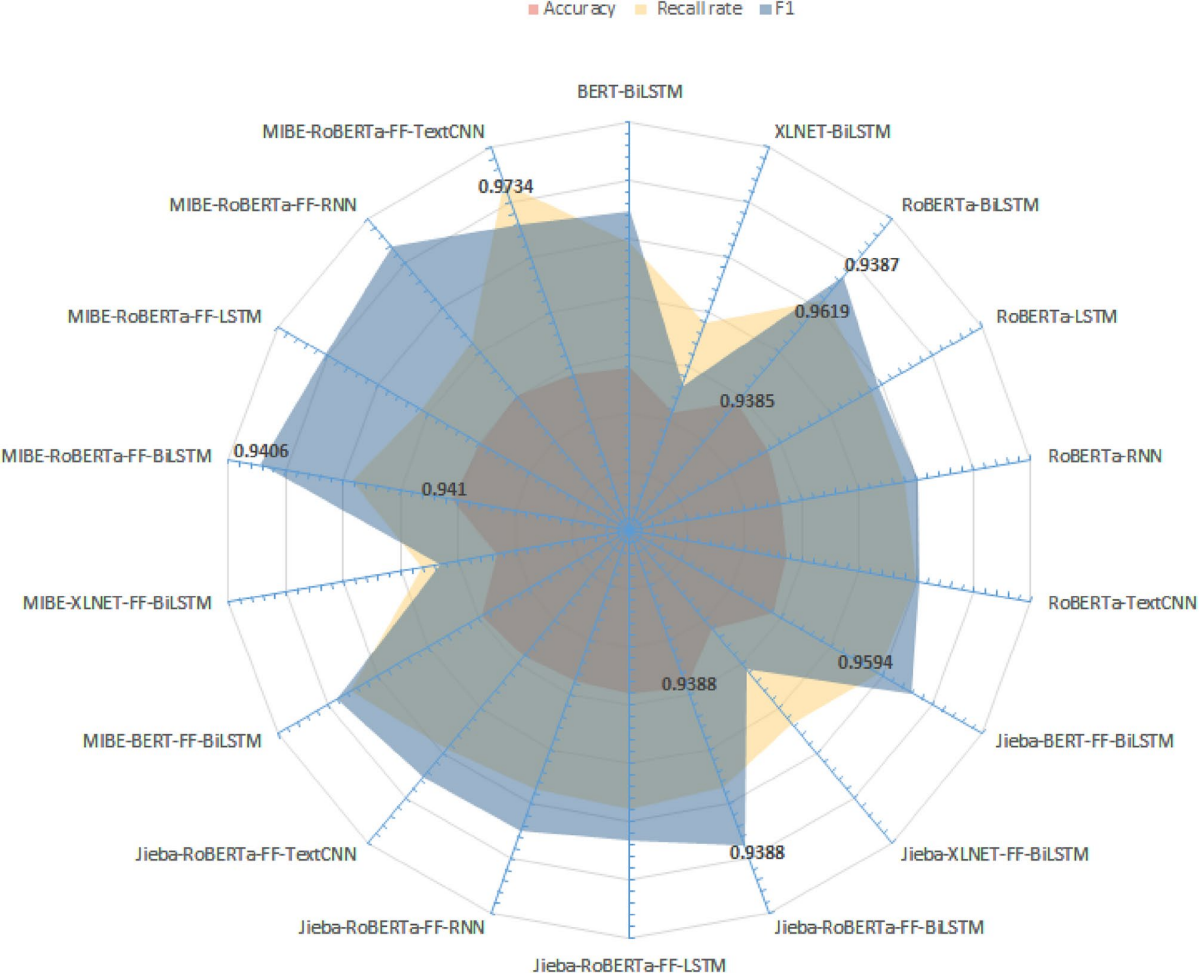
Comprehensive statistics of the performance of the sentiment analysis model, respectively.

In order to visually compare the performance of each comparative model, this paper, based on Table 3, draws Fig. 7 (performance statistics of mainstream baseline model for sentiment analysis), Fig. 8 (performance statistics of mainstream baseline model with the introduction of the jieba lexicon and the FF layer), Fig. 9 (performance statistics of mainstream baseline model with the introduction of the MIBE-based lexicon and the FF layer), and Fig. 10 (comprehensive statistics of the performance of the sentiment analysis model), respectively.

Based on Fig. 7, it can be found that among the mainstream baseline models, the RoBERTa-BiLSTM model has the best performance, with accuracy, recall and F1 value exceeding 93.85%, which indicates that the RoBERTa pre-trained model is able to adequately extract the semantic and structural information of the danmaku text, and then using the bidirectional sequence modeling capability of the BiLSTM model, it can effectively capture the contextual information of danmaku text as well as fitting the textual characteristics of danmaku text with varying length and linguistic diversity, better understanding and modeling the dependency relationships in danmaku text, and improving the model's ability to classify emotional tendencies. The BERT-BiLSTM model performs slightly lower than the RoBERTa-BiLSTM model on this task, probably because the RoBERTa model uses a larger dataset, larger batch size, higher learning rate, and better masking strategy compared to the BERT model during the pre-training process, which allows it to show more robustness and higher optimization level. XLNET-BiLSTM has the worst performance on this task, probably because XLNET adopts an improved autoregressive training approach, which may lead to its performance on sentiment classification is not as good as that of the BERT and RoBERTa models that adopt an autocoding training approach; and chinese-roberta-wwm-ext and chinese-bert-wwm-ext are models specifically pre-trained for use on Chinese text, their lexical and grammatical comprehension is more powerful on Chinese text, which is more suitable for the Chinese sentiment classification task. RoBERTa-TextCNN model achieved a good performance on this task table, but there is a gap with BERT-BiLSTM and RoBERTa-BiLSTM model, probably because the TextCNN model is to use convolution kernel sliding on the text sequence to capture the local information, and get the global information through the pooling layer, and its ability to capture the global long-distance dependence is relatively weak, and it can not effectively deal with the characteristics of the danmaku text of varying lengths and linguistic diversity, and it may be the problem of information loss or noise interference. RoBERTa-RNN and RoBERTa-LSTM models perform slightly worse on this task, probably because RNN has a weak ability to capture the information before and after the danmaku text sequence and is prone to gradient vanishing and gradient explosion problems; LSTM model has a lower information utilization rate compared to BiLSTM model, which relies on unidirectional information transfer only, and some important contextual information is easy to be ignored, and the ability to capture bidirectional dependencies of the danmaku text is slightly weak. The neural network and machine learning methods without using pre-trained models performed the worst, with the overall performance far lower than the methods using pre-trained models. Among them, the SVM model performed relatively well, with the accuracy, recall and F1 values all exceeding 88.50%. The model had a strong generalization ability in dealing with binary classification problems, but it focused on the selection and representation of features. The semantic features of danmaku texts were complex, which might exceed the model’s processing ability. The BiLSTM model performed second, and only learned simple temporal information without the support of pre-trained models. It was difficult to learn the deep and rich linguistic knowledge of danmaku texts. The BernoulliNB model performed the worst, as it required binarization of the data, which resulted in some information loss and affected the quality and integrity of the data.

Based on Fig. 8, it can be found that after the introduction of jieba word-splitting lexicon and embedding FF feature fusion layer in the mainstream baseline model, the performance of each model is improved, especially the F1 values of XLNET-BiLSTM, RoBERTa-LSTM, RoBERTa-RNN, and RoBERTa-TextCNN are all increased by more than 0.1%. It indicates that the introduction of jieba lexicon can cut Chinese danmaku text into more reasonable words, reduce noise and ambiguity, and improve the quality of word embedding. The FF feature fusion layer is able to average the feature encoding of the words in each word to get the word encoding that retains the semantic information of the word, which makes Chinese word splitting meaningful, and is also helpful to eliminate the effect of multiple meanings of words, express more accurate information, and enhance the semantic comprehension and generalization ability of the model.

Based on Fig. 9, it can be found that after adding MIBE neologism recognition to the model in Fig. 7, the performance of each model is improved, especially the accuracy and F1 value of RoBERTa-FF-BiLSTM, RoBERTa-FF-LSTM, and RoBERTa-FF-RNN are increased by about 0.2%. It shows that the MIBE neologism recognition algorithm based on MIBE can effectively discover neologisms in danmaku text, and the introduction of jieba can enrich its participle thesaurus and improve the accuracy of Chinese participle, which in turn improves the performance of the model. Therefore, it is also demonstrated that there are a large number of non-standard and creative web-popular neologisms in danmaku text, which can negatively affect the model's semantic comprehension and sentiment categorization ability if they are not recognized.

Based on Fig. 10, comprehensively, the MIBE-RoBERTa-FF-BiLSTM model proposed in this paper has the best overall performance in the task of sentiment categorization of Chinese danmaku text in video, with an accuracy rate of 94.10%, a recall rate of 95.84%, and an F1 value of 94.06%. It indicates that the MIBE-RoBERTa-FF-BiLSTM model can effectively deal with the informal language of danmaku text in this task, and can effectively discover new words in danmaku text, fully extract the semantic and structural information of danmaku text, and learn the word information, character information, and contextual information of danmaku text, so as to better understand and model the semantic and dependency relationships in danmaku text, and improve the model's ability to classify emotional tendencies and generalization.

## Discussions

This paper proposes to classify the sentiment of danmaku texts based on the MIBE-RoBERTa-FF-BiLSTM model, and adopts the neologism recognition algorithm based on mutual information (MI) and branchentropy (BE), which can effectively discover the non-regular popular neologisms with more than three elements in danmaku text, and introduce the jieba lexical lexicon, which can slice the text into more reasonable words, such as "爷青回", "蚌埠住了", etc. It reduces noise and ambiguity, improves the quality of word embeddings, is more efficient and adaptable to the characteristics and variations of danmaku texts compared to traditional lexicons, and is able to capture fresh and interesting expressions in the text, which solves RQ2. Based on Maslow's hierarchy of needs theory, this paper argues that danmaku text emotion is jointly generated by individual needs and external stimuli. By parsing the hierarchy of needs in danmaku and combining it with the video content, it improves the reasonableness and consistency of the emotion annotation, which is more reflective of the psychological state and motivation of the danmaku users than the traditional annotation, reduces the difficulty of the annotation, and solves the RQ1. This paper uses RoBERTa-FF-BiLSTM model to learn the semantic features of danmaku texts, and the pre-trained model based on RoBERTa is able to fully extract the semantic and structural information of danmaku text, learn deeper and richer linguistic knowledge than traditional machine learning or shallow neural network methods, improve the model's ability to generalize on small-scale data, and solve the RQ3; Based on the feature fusion layer the feature encoding of the words in each word after word splitting is averaged and then filled into the position of the original word, compared with the traditional word encoding method, it is able to obtain the word encoding that retains the semantic information of the word, eliminates the effect of multiple meanings of the word, and solves the RQ4; Based on the BiLSTM model to capture the contextual information of danmaku text, it efficiently fits the textual characteristics of danmaku text of varying lengths and linguistic diversity, and is able to adapt to danmaku text of different lengths and styles compared to traditional convolutional neural network or recurrent neural network approaches.

However, the study of the text has the following limitations and challenges:

1. The danmaku text contains a large number of popular new words on the Internet, such as self-made words, abbreviations, interactive words, etc. These new words increase the difficulty of semantic understanding and emotional expression of the danmaku text. This paper uses a danmaku new word recognition algorithm based on MIBE, which can automatically discover irregular popular words in the danmaku text, but still needs manual semantic understanding and evaluation in combination with the context and video content, judging the quality of the new words, eliminating invalid words, and the degree of automation is limited. The manual review process is not only time-consuming and labor-intensive, but also may have subjective bias and inconsistency, affecting the quality and reliability of the new word dictionary.
2. This paper only considers the danmaku text content, ignoring the semantic information of the danmaku video content, and the understanding and evaluation of the danmaku’s emotional expression is relatively single. The danmaku text and video content are interrelated and influenced by each other. The emotional tendency and expression of the danmaku users are often stimulated and guided by the video content. Simply analyzing the danmaku text content may ignore some important emotional information and contextual information, resulting in the sentiment analysis results not being accurate and comprehensive enough.
3. In the comparative experiment, the training paradigm of “pre-trained model + neural network classifier” was used. Although it achieved high performance, there were also some problems. The recall rate of the model under this paradigm was higher than the accuracy and F1 values, indicating that the model’s prediction results and true labels had deviations, and the model was more inclined to predict negative cases as positive cases, resulting in positive case preference problems.

In the future, the following aspects can be considered for optimization and improvement:

1. We will perform topic identification on the danmaku new words identified by the MIBE algorithm, design corresponding prompts, and use large language models such as GTP4, Llama2, ChatGLM3, etc. to perform semantic analysis and quality evaluation on the danmaku new words more logically and efficiently, filter out meaningful and useful new words, automatically construct the danmaku new word dictionary, and reduce the workload and error of manual review.
2. We will use multimodal representation methods such as CLIP to extract and fuse features of the danmaku text and video content, capture the interactive emotional information between the danmaku text and video content, and understand the danmaku’s emotional expression more comprehensively and accurately.
3. We will design a text quality evaluation and error correction model of “pre-trained model (T5, MacBERT, etc.) + external knowledge base”, try to evaluate and score the danmaku texts with positive and negative emotions, clean, standardize, correct, etc. the texts with low quality, improve the quality of the texts, make the texts with positive and negative emotions more close in quality, thereby reducing the model’s positive case preference, and improve the model’s prediction performance and generalization ability.

## Conclusion

This paper presents a video danmaku sentiment analysis method based on MIBE-RoBERTa-FF-BiLSTM. It employs Maslow's Hierarchy of Needs theory to enhance sentiment annotation consistency, effectively identifies non-standard web-popular neologisms in danmaku text, and extracts semantic and structural information comprehensively. By learning word, character, and context information, the model better understands and models semantic and dependency relationships in danmaku text. It outperforms mainstream models in video danmaku sentiment classification. This research method offers a novel perspective on video danmaku sentiment analysis, serving as a valuable reference for related fields.

## Data Availability

This paper collect danmaku texts from Bilibili through web crawler, and construct a "Bilibili Must-Watch List and Top Video Danmaku Sentiment Dataset" with a total of 20,000 pieces of data. The datasets and codes generated during the current study are available from the corresponding author on reasonable request.
